# A86 DIETARY BIOMARKERS OF ULTRA-PROCESSED FOODS: A NARRATIVE REVIEW

**DOI:** 10.1093/jcag/gwae059.086

**Published:** 2025-02-10

**Authors:** T S Armstrong, K Chappell, L J Ajibulu, K Wong

**Affiliations:** University of Alberta Faculty of Medicine & Dentistry, Edmonton, AB, Canada; University of Alberta Faculty of Medicine & Dentistry, Edmonton, AB, Canada; Medicine, University of Alberta Faculty of Medicine & Dentistry, Edmonton, AB, Canada; Medicine, University of Alberta Faculty of Medicine & Dentistry, Edmonton, AB, Canada

## Abstract

**Background:**

**The rapidly increasing prevalence of ultra-processed foods (UPFs) in global diets necessitates a more comprehensive understanding of this food group’s effects on health and disease. Nutritional biomarkers are critical for developing this understanding, as they provide an objective assessment of the body’s response to UPF intake.**

**Aims:**

This study aimed to assemble a comprehensive list of the current clinical biomarkers of ultra-processed foods, then categorize them and evaluate their links to disease states and health.

**Methods:**

This narrative review assessed the findings of eight studies, all of which focused on the biomarkers of ultra-processed foods and were identified via a systematic search of the literature that included stringent criteria. Pertinent information was extracted and summarized from these studies, with a primary focus on the biomarkers identified and an exploration of some of these biomarkers’ connections with disease.

**Results:**

Based on the data extracted from the eight included studies, we categorized ultra-processed food biomarkers into organic acids (including amino acids), lipids/lipid-like molecules, xenobiotic food components (specifically associated with UPFs), and other molecular compounds (dietary oxysterols, nucleotides, proteins, etc). Via correlations between these four categories and diseases, biomarkers of ultra-processed foods were found to be associated with inflammatory mechanisms, as well as a variety of pathologies including intestinal disorders, atherosclerosis, chronic kidney disease, and others.

**Conclusions:**

The usefulness of our review lies in its narrative format and succinct accumulation of information pertinent to ultra-processed food analysis, while also reinforcing the detrimental impacts of this food group on health and nutrition. These findings emphasized the importance of future studies concerning ultra-processed foods and food processing techniques, while providing a succinct summary of the current biomarkers of ultra-processed foods in relevant literature.

Funding Agencies:

